# Effectiveness of combined antithrombin and thrombomodulin therapy on in-hospital mortality in mechanically ventilated septic patients with disseminated intravascular coagulation

**DOI:** 10.1038/s41598-020-61809-2

**Published:** 2020-03-17

**Authors:** Takeshi Umegaki, Susumu Kunisawa, Kota Nishimoto, Takahiko Kamibayashi, Yuichi Imanaka

**Affiliations:** 1 Department of Anesthesiology, Kansai Medical University Hospital, Osaka, Japan; 2 Department of Healthcare Economics and Quality Management, Graduate School of Medicine, Kyoto University, Kyoto, Japan

**Keywords:** Outcomes research, Chemical physics

## Abstract

Septic patients can develop disseminated intravascular coagulation (DIC), which is characterized by systemic blood coagulation and an increased risk of life-threatening haemorrhage. Although antithrombin (AT) and thrombomodulin (TM) combination anticoagulant therapy is frequently used to treat septic patients with DIC in Japan, its effectiveness in improving patient outcomes remains unclear. In this large-scale multicentre retrospective study of adult septic patients with DIC treated at Japanese hospitals between February 2010 and March 2016, we compared in-hospital mortality between AT monotherapy and AT + TM combination therapy. We performed logistic regression analysis with in-hospital mortality as the dependent variable and anticoagulant therapy as the main independent variable of interest. Covariates included patient demographics, disease severity, and body surface area. The AT group and AT + TM group comprised 1,017 patients from 352 hospitals and 1,205 patients from 349 hospitals, respectively. AT + TM combination therapy was not significantly associated with lower mortality when compared with AT monotherapy (odds ratio: 0.97, 95% confidence interval: 0.78–1.21; *P *= 0.81). AT + TM combination therapy was also not superior to AT monotherapy in reducing mechanical ventilation or hospitalization durations. Despite its widespread use for treating sepsis with DIC, AT + TM combination therapy is not more effective in improving prognoses than the simpler AT monotherapy.

## Introduction

Sepsis can lead to disseminated intravascular coagulation (DIC), which is a serious condition characterized by widespread coagulation throughout a patient’s bloodstream^1,2^. The rapid depletion of blood coagulation factors in these patients increases the risk of potentially fatal haemorrhage. In Japan, this condition was responsible for 10,318 deaths per 100,000 population in 2018, which was comparable to the mortality rates of oesophageal tumours and malignant lymphomas^3^.

Antithrombin (AT) and thrombomodulin (TM) are frequently administered together as anticoagulant therapy to treat septic patients with DIC in Japan. In a meta-analysis of sepsis-induced DIC, Yatabe *et al*. reported that AT use is a key factor in DIC resolution^4^. A phase III trial conducted between 2000 and 2005 found that TM was not significantly associated with improvements in 28-day mortality when compared with heparin, but that its use significantly improved DIC^5^. Similarly, the multinational, multicentre SCARLET randomized clinical trial reported that TM use in patients with sepsis-associated coagulopathy was not significantly associated with reduced mortality^6^. In Japan, a multicentre post-marketing survey conducted between 2014 and 2016 showed that approximately half of 510 sepsis-associated DIC patients were treated with AT + TM combination therapy despite the lack of evidence to support its use^7^. However, little is known about the effectiveness of AT + TM combination therapy on mortality in septic patients with DIC, especially in more severe cases that require mechanical ventilation due to respiratory dysfunction.

Although randomized controlled trials are the gold standard for examining the effects of therapy on clinical outcomes, they are limited by logistical, ethical, and financial constraints. Administrative data, or insurance claims data, are produced by hospitals for the purpose of reimbursements from insurers, and provide a viable alternative that allows analyses of large sample sizes with relative ease. Due to the wide range of available information, administrative data are increasingly applied to healthcare research. Many acute care hospitals in Japan use the Diagnosis Procedure Combination (DPC) patient case-mix system to specify the level of reimbursements for each hospitalization episode. This information includes patient baseline characteristics, medical tests, medications, and procedures. DPC data also contain information on the use of mechanical ventilation, renal replacement therapy, and vasoactive agents, which are associated with increased mortality^8–13^.

In this study, we used Japanese DPC administrative data to comparatively examine in-hospital mortality between AT monotherapy and AT + TM combination therapy in mechanically ventilated septic patients with DIC.

## Methods

### Study design and data source

We conducted a retrospective multicentre cohort study of septic patients with DIC requiring mechanical ventilation between February 1, 2010 and March 31, 2016 at acute care hospitals located throughout Japan. We compared the characteristics between patients who had been administered AT monotherapy (AT group) and patients who had been administered AT + TM combination therapy (AT + TM group).

The data source was a DPC database comprising clinical information and healthcare claims data collected by the government-funded DPC Research Group, which is tasked with improving healthcare in Japan through analyses of DPC data. The data set included patient demographics (e.g., sex, height, and weight), primary and secondary diagnoses, comorbidities, surgeries, disease severity, and specific treatments (e.g., mechanical ventilation). The study was approved by the institutional ethics committee of Kansai Medical University Hospital (Approval Number: 2017025). All methods were performed in accordance with the Ethical Guidelines for Medical and Health Research Involving Human Subjects stipulated by the Japanese Ministry of Health, Labour and Welfare. As the database comprised anonymized claims information with no personally identifiable information, the ethics committee waived the need for informed consent.

### Patient selection

Patients diagnosed with sepsis were identified using the relevant International Classification of Diseases, 10th Revision (ICD-10) codes listed in Table 1. DIC was identified using the ICD-10 code D65. The dates of intensive care unit admission and discharge were determined according to the specific Japanese hospitalization codes provided in the administrative data. The use of mechanical ventilation was identified from the corresponding Japanese procedural codes. Cases with non-invasive positive pressure ventilation were not included in the analysis.

**Table 1:** ICD-10 codes used for identification of septic patients and acute organ dysfunction.

Condition	ICD-10 code
Salmonella septicemia	A02.1
Septicemic plague	A20.7
Anthrax septicemia	A22.7
Melioidosis	A24.1
Erysipelothrix septicemia	A26.7
Extraintestinal yersiniosis	A28.2
Listerial septicemia	A32.7
Meningococcal bacteremia	A39.4
Streptococcal septicemia	A40
Other septicemia	A41
Actinomycotic septicemia	A42.7
Disseminated herpesviral disease	B00.7
Viremia	B34.9
Candidal septicemia	B37.7
Pericarditis	I30.1
Endocarditis	I33.0
Infective myocarditis	I40.0
Arteritis	I77.6
Streptococcal pharyngitis	J02.0

Abbreviation: ICD-10, International Classification of Diseases, Tenth Revision.

### Patient characteristics

Information on the following patient baseline characteristics were collected: age, sex, height, weight, primary diagnosis, activities of daily living (ADL) score at admission and discharge, Charlson comorbidity index, number of organ failures, renal replacement therapy, and blood product transfusions.

ADL scoring was based on the following 10 items: feeding (0–2 points), transferring (0–3 points), grooming (0–1 point), toileting (0–2 points), bathing (0–1 point), walking on level ground (0–3 points), climbing stairs (0–2 points), dressing (0–2 points), bowel continence (0–2 points), and urinary continence (0–2 points). The maximum score was 20 points. The Charlson comorbidity index is widely used as a severity score to predict one-year mortality^14^. For this study, we used a version of the comorbidity index designed for applications in administrative data^15^. Each patient’s height and weight were used to calculate their body surface area based on the DuBois formula ([Weight in kg ^0.425^ × Height in cm ^0.725^] × 0.007184); body surface area was used as an indicator of physical constitution. Organ failure was identified based on the criteria proposed by Angus *et al*., which used ICD-9 codes^16^. As Japanese hospitals use ICD-10 codes instead of ICD-9 codes, these codes were first recoded to ICD-10 codes before being used to identify diagnoses and organ failure (Table 2). Renal replacement therapy included both intermittent and continuous renal replacement therapies. Blood product transfusions included transfusions of red blood cells, fresh frozen plasma, and platelets.

**Table 2:** Recoding of ICD-9-CM codes into the corresponding ICD-10 codes.

Diagnostic category	ICD-9-CM code	ICD-10 code
**Cardiovascular dysfunction**		
Shock without trauma	785.5	A419
		A483
		R570
		R571
		R578
		R579
Hypotension	458	I959
**Neurologic dysfunction**		
Encephalopathy	348.3	F058
		G934
		G938
		I672
		I674
		I678
		K729
		K868
		G948
Transient organic psychosis	293	F069
Anoxic brain damage	348.1	G931
**Hematologic dysfunction**		
Secondary thrombocytopenia	287.4	D695
Thrombocytopenia, unspecified	287.5	D696
Other/unspecified coagulation defect	286.9	D65
Defibrination syndrome	286.6	D65
**Hepatic dysfunction**		
Acute and subacute necrosis of liver	570	K729
Hepatic infarction	573.4	K763
**Renal dysfunction**		
Acute renal failure	584	N179

Abbreviations: ICD-9-CM, International Classification of Diseases, Ninth Revision, Clinical Modification; ICD-10, International Classification of Diseases, Tenth Revision.

### Outcome measures

The primary outcome measure was in-hospital mortality. The secondary outcome measures were the length of intensive care unit stay, length of hospital stay from intensive care unit admission, and duration of mechanical ventilation.

### Statistical analysis

All continuous variables were calculated as means and standard deviations, and all categorical variables were calculated as percentages. We employed Student’s *t* test and the chi-squared test to compare continuous and categorical variables, respectively, between the AT group and the AT + TM group. Univariate analyses were used to identify patient characteristics with a significant association with in-hospital mortality; these characteristics were included as covariates in a multivariable logistic regression analysis with in-hospital mortality as the dependent variable and the use of AT + TM combination therapy (versus AT monotherapy) as the main independent variable of interest. Other independent variables were included if their *P* values were below 0.2 in the univariate analyses. The probability of TM use was calculated based on its administration rate for septic patients with DIC in each hospital, and was used as an instrumental variable. The odds ratios and 95% confidence intervals for the independent variables were calculated. In addition, Kaplan-Meier survival curves were plotted to examine the differences in survival between the AT group and the AT + TM group. The Mantel-Haenszel test was used to analyze therapy-specific mortality between men and women.

*P* values lower than 0.05 were considered to be statistically significant. All analyses were performed using SPSS Version 25.0 (IBM Japan, Ltd., Tokyo, Japan).

## Results

### Patient characteristics

The patient characteristics of the study sample (n = 2,222 patients from 467 hospitals) are presented in Table 3. The AT group and AT + TM group comprised 1,017 patients from 352 hospitals and 1,205 patients from 349 hospitals, respectively. The mean ages of the patients in the AT and AT + TM groups were 68.9 years and 70.3 years (*P* = 0.02), respectively. There were also significant differences between the groups in sex (*P* = 0.03), height (*P* = 0.001), body surface area (*P* < 0.01), and ADL score at discharge (*P* < 0.001). The use of renal replacement therapy was significantly higher (*P* < 0.001) in the AT + TM group (72.4%) than in the AT group (59.1%). In contrast, the AT + TM group had significantly lower blood product transfusions than the AT group. The duration of AT administration in the AT + TM group (mean±standard deviation: 4.3 ± 3.7 days) was significantly longer (*P* < 0.001) than in the AT group (3.6 ± 3.2 days). TM was administered to patients in the AT + TM group for approximately one week.

**Table 3:** Characteristics of mechanically ventilated septic patients with DIC in the AT and AT + TM groups (n = 2,222).

	AT group (n = 1,017)	AT + TM group (n = 1,205)	*P* value
**Patient characteristics**			
Age (years)	68.9 ± 14.1	70.3 ± 13.4	0.02
Male	63.8%	59.2%	0.03
Height (cm)	147.7 ± 42.8	153.3 ± 30.8	0.001
Weight (kg)	54.6 ± 27.2	54.8 ± 16.3	0.85
Body mass index (kg/m^2^)	22.4 ± 9.5	22.2 ± 5.0	0.47
Body surface area (m^2^)	1.4 ± 0.5	1.5 ± 0.4	<0.01
ADL score at admission	8.5 ± 9.0	7.9 ± 9.0	0.16
ADL score at discharge	12.4 ± 8.7	10.3 ± 8.8	<0.001
Charlson comorbidity index	1.5 ± 1.8	1.4 ± 1.7	0.13
Number of organ failures			
2	39.6%	35.6%	0.14
3	43.0%	46.2%	
≥4	17.4%	18.2%	
**Processes of care**			
Duration of AT use (days)	3.6 ± 3.2	4.3 ± 3.7	<0.001
Duration of TM use (days)	—	6.7 ± 4.9	<0.001
Renal replacement therapy	59.1%	72.4%	<0.001
Blood product transfusion (mL)			
Red blood cells	630.4 ± 554.4	364.2 ± 176.3	<0.001
Fresh frozen plasma	1622.4 ± 1856.2	878.4 ± 871.8	<0.001
Platelets	842.6 ± 482.1	240.8 ± 79.4	<0.01

Values are presented as mean ± standard deviation for continuous variables and as a percentage for categorical variables.

Abbreviations: ADL, activities of daily living; AT, antithrombin; DIC, disseminated intravascular coagulation; TM, thrombomodulin.

### Anticoagulant therapy and outcome measures

Table 4 shows the differences in outcomes between the 2 groups. The univariate analysis found no significant difference (*P* = 0.46) in in-hospital mortality between the AT + TM group (54.8%) and the AT group (53.2%). There was no significant difference (*P* = 0.95) in the length of intensive care unit stay between the AT + TM group (mean±standard deviation: 8.9 ± 5.5 days) and the AT group (9.0 ± 5.5 days). Similarly, there was no significant difference (*P* = 0.53) in the length of hospital stay between the AT + TM group (mean ± standard deviation: 60.0 ± 54.6 days) and the AT group (58.5 ± 54.9 days). The duration of mechanical ventilation in the AT + TM group (mean±standard deviation: 16.4 ± 14.4 days) was significantly longer (*P* = 0.02) than in the AT group (15.0 ± 14.4 days).

**Table 4:** Outcomes in mechanically ventilated septic patients with DIC in the AT and AT + TM groups (n = 2,222).

	AT group (n = 1,017)	AT + TM group (n = 1,205)	*P* value
**Outcomes**			
In-hospital mortality	53.2%	54.8%	0.46
Intensive care unit stay (days)	9.0 ± 5.5	8.9 ± 5.5	0.95
Hospital stay (days)	58.5 ± 54.9	60.0 ± 54.6	0.53
Duration of mechanical ventilation (days)	15.0 ± 14.4	16.4 ± 14.4	0.02

Values are presented as mean ± standard deviation for continuous variables and as a percentage for categorical variables.

Abbreviations: AT, antithrombin; DIC, disseminated intravascular coagulation; TM, thrombomodulin.

**Figure 1:**
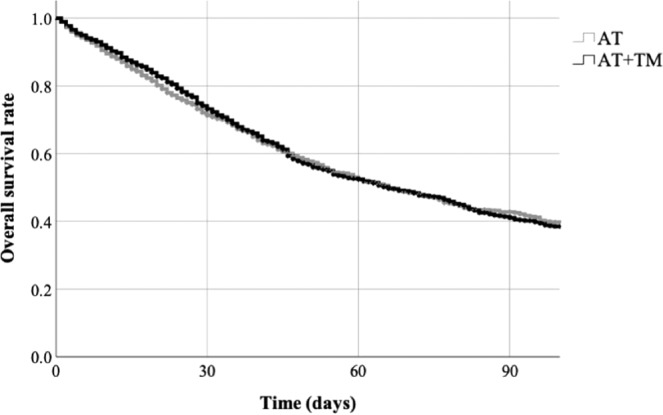
Kaplan-Meier survival curves of the antithrombin (AT) monotherapy group and the antithrombin and thrombomodulin (AT + TM) combination therapy group. There was no significant difference in survival between the groups (*P* = 0.94).

The results of the logistic regression and instrumental variable analysis for in-hospital mortality are presented in Table 5. The AT + TM group was not significantly associated with a lower risk of in-hospital mortality (odds ratio: 0.97; 95% confidence interval: 0.78–1.21; *P* = 0.81) relative to the AT group. However, men had a significantly higher risk of in-hospital mortality (odds ratio: 1.58; 95% confidence interval: 1.29–1.95; *P* < 0.001) relative to women. As shown in Fig. 1, there was no significant difference in the Kaplan-Meier survival curves between the groups (*P* = 0.94). Table 6 shows the numbers of survivors and non-survivors in the AT and AT + TM groups according to sex. Both therapies were not significantly associated with in-hospital mortality regardless of sex (*P* = 0.36).

**Table 5:** Results of logistic regression with an instrumental variable analysis for in-hospital mortality (n = 2,222).

	Odds ratio	95% CI	*P* value
AT + TM (ref: AT only)	0.97	0.78–1.21	0.81
Age	1.02	1.01–1.03	<0.001
Male (ref. female)	1.58	1.29–1.95	<0.001
Body surface area	0.68	0.52–0.89	<0.01
Charlson comorbidity index	1.14	1.08–1.21	<0.001
Number of organ failures (ref. ^2^)			
3	1.08	0.87–1.35	0.47
≥4	1.54	1.13–2.08	<0.01
ADL score at admission	1.00	0.99–1.01	0.65
Duration of mechanical ventilation	1.01	1.01–1.02	<0.001
Renal replacement therapy	1.50	1.20–1.88	<0.001

Abbreviations: ADL, activities of daily living; AT, antithrombin; CI, confidence intervals; TM, thrombomodulin.

**Table 6:** Associations between the anticoagulant therapies and in-hospital mortality according to sex.

	Male		Female		*P* value
	Survivors	Non-survivors	Survivors	Non-survivors	
AT group	288	361	188	180	0.36
AT + TM group	287	426	258	234	

Abbreviations: AT, antithrombin; TM, thrombomodulin.

## Discussion

In this large-scale multicentre analysis of administrative data, we comparatively examined in-hospital mortality between AT monotherapy and AT + TM combination therapy in mechanically ventilated septic patients with DIC. The study showed that AT + TM combination therapy was not significantly associated with reduced in-hospital mortality when compared with AT monotherapy.

In another analysis of Japanese administrative data, Murata *et al*. similarly found no significant differences in in-hospital mortality between AT and TM in patients with infection-associated DIC^17^. Despite the differences in DIC aetiology, the findings of our study corroborate their conclusions. However, it should be noted that the length of hospital stay in our study subjects was approximately 20 days longer than that of the subjects in Murata *et al*.^17^. This disparity could be due to our focus on more severe patients who required mechanical ventilation for respiratory dysfunction.

In contrast, a single-centre observational study of patients with severe sepsis and DIC reported that combined AT and TM resulted in lower 28-day mortality rates than AT alone^18^, and other studies have produced similar findings on 28-day mortality in rats^19,20^. Our analysis therefore contradicts the findings of these studies. However, the results of single-centre studies should be interpreted with caution as they are more susceptible to the effects of hospital-specific characteristics. In addition, our analysis was limited to patients who required mechanical ventilation, and may represent severe cases who are less responsive to treatment.

A retrospective study used propensity-score matching and instrumental variable analyses to examine mechanically ventilated patients with sepsis and DIC after intestinal perforation, and found no association between TM administration and 28-day mortality^21^. Similarly, other studies using DPC databases have also reported no associations between anticoagulant therapies and mortality in septic patients with DIC^17,21^. TM administration in mechanically ventilated patients with acute respiratory distress syndrome and DIC has also been found to improve oxygenation^22^. However, our analysis did not find any clinical advantages in using TM for mechanically ventilated patients with sepsis and DIC. In addition, we could not conduct a detailed analysis of respiratory function due to the limitations of the DPC database. Iba *et al*. reported a significant improvement in 28-day mortality following AT + TM combination therapy in patients with sepsis-associated DIC^7^. Although our study was characterized by substantially longer observation periods and more subject hospitals, the differences between our findings and those of Iba *et al*.^7^ underscore the need for further research to explore the merits of AT + TM combination therapy.

Our analysis used body surface area as an indicator of physical constitution. Studies of critically ill patients generally use body weight or body mass index as they can be indicative of the amount of medication administered. However, body surface area is widely used for analysing cardiac patients as it mitigates variations in patient size and abnormal adipose mass. The mean body surface area of our study subjects was 1.4–1.5, indicating that Japanese patients are generally smaller than those in the US^23^. Body surface area may be a more useful characterization than body weight or body mass index of patients in large-scale studies in the Japanese healthcare setting. In addition, patients in the AT group had marginally higher ADL scores at discharge than patients in the AT + TM group (Appendix 1). We posit that the poorer ADL scores in the latter group may be influenced by the patients’ older age and higher number of organ failures, but further studies are needed to explore this possibility.

This study has several limitations. First, there was a lack of information on each patient’s blood examination results. Physiological data are not included in the DPC database, and we were unable to examine platelet count, prothrombin time, white blood cell count, fibrin/fibrinogen degradation products, pathogens, sources of infection, and inflammatory markers. Accordingly, our analysis depended on the accuracy of the DPC records, which may be vulnerable to upcoding or miscoding. However, these records are sent to insurers for reimbursements, and are therefore subject to stringent regulations and assessments. Therefore, the number of errors is unlikely to be high. Second, our identification of septic patients using ICD-10 codes may have omitted some cases of sepsis that were not covered by the codes. However, our identification criteria included the ICD-10 code A41, which refers to cases of sepsis without an identified causative pathogen. We therefore believe that the majority of septic patients were identified using our criteria, and any missed cases would be unlikely to influence our findings. Third, there was a lack of physiological and radiographic data, such as oxygen saturation indices and chest radiographs. As a consequence, we were unable to determine the level of respiratory dysfunction in patients who used mechanical ventilation. Nevertheless, we consider that the majority of septic patients with DIC who used mechanical ventilation had severe oxygen desaturation and needed mechanical support to sustain gas exchange. Accordingly, mechanical ventilation was used as an indicator of respiratory dysfunction. Fourth, we were unable to determine the reasons for the lower quantity of blood product transfusions in the AT + TM group. It is possible that physicians may have chosen to administer AT only instead of AT + TM to patients with a perceived higher risk of haemorrhage. This may have increased the proportion of patients with lower haemorrhage risk in the AT + TM group, which resulted in the lower quantity of blood product transfusions. However, additional studies using medical records are needed to examine the clinical reasons for this difference. Fifth, we were unable to identify the causes of death and adverse events from anticoagulation therapy. The main adverse effect of anticoagulant therapy would be excessive bleeding, but a previous study found no significant difference in bleeding events between AT monotherapy and AT + TM combination therapy^7^. Finally, it is possible that there are other factors that affect in-hospital mortality (e.g., medical and surgical history) that were not included in our covariates. This study employed a retrospective design using claims data, which limited our access to detailed clinical information. The Sequential Organ Failure Assessment score was introduced into the DPC system in 2018, and a combination of this score and the Charlson comorbidity index may enable evaluations of the severity of critical illnesses and comorbidities in future studies.

This study is, to the best of our knowledge, the first to observe that AT + TM combination therapy is not superior to AT monotherapy with regard to in-hospital mortality, hospital stay, and mechanical ventilation duration in mechanically ventilated septic patients with DIC. These findings indicate a need to re-examine the use of this therapy, and presents an opportunity to streamline therapeutic strategies for these patients.

## Supplementary information

Supplementary information

## Data Availability

The data set used in this study is available from the corresponding author on reasonable request.
