# Supplementary material for: Effectiveness of combined antithrombin and thrombomodulin therapy on in-hospital mortality in mechanically ventilated septic patients with disseminated intravascular coagulation
Source: Sci Rep. 2020 Mar 17;10:4874. doi: 10.1038/s41598-020-61809-2 (PMC7078266; doi:10.1038/s41598-020-61809-2)
Supplement: Supplementary file 1 — Supplementary information [file 41598_2020_61809_MOESM1_ESM.docx]

# Effectiveness of combined antithrombin and thrombomodulin therapy on in-hospital mortality in mechanically ventilated septic patients with disseminated intravascular coagulation

Takeshi Umegaki^1*^, Susumu Kunisawa^2^, Kota Nishimoto^1^, Takahiko Kamibayashi^1^, Yuichi Imanaka^2^

Authors’ affiliations:

^1^ Department of Anesthesiology, Kansai Medical University Hospital, Osaka, Japan

^2^ Department of Healthcare Economics and Quality Management, Graduate School of Medicine, Kyoto University, Kyoto, Japan

*Corresponding author: Takeshi Umegaki, MD, PhD

Department of Anesthesiology, Kansai Medical University Hospital,

2-3-1 Shin-machi, Hirakata, Osaka 573-1191, Japan

Tel: +81-72-804-0100

Fax: +81-72-804-2785

E-mail: umegakit@hirakata.kmu.ac.jp

Corresponding author after publication: Yuichi Imanaka, MD, PhD

Department of Healthcare Economics and Quality Management, Graduate School of Medicine, Kyoto University, Yoshida Konoe-cho, Sakyo-ku, Kyoto 606-8501, Japan

Tel: +81-75-753-4454

Fax: +81-75-753-4455

E-mail: imanaka-y@umin.net

**Appendix 1.** Activity-specific ADL scores at admission and discharge (n=2,222).

| **Activity** | **AT group**  **(n=1,017)** | **AT+TM group**  **(n=1,205)** | ***P* value** |
| --- | --- | --- | --- |
| At admission, Score (IQR) |  |  |  |
| Feeding | 1(0-2) | 1(0-2) | 0.29 |
| Transferring | 0(0-3) | 0(0-3) | 0.26 |
| Grooming | 0(0-1) | 0(0-1) | 0.38 |
| Toileting | 0(0-2) | 0(0-2) | 0.21 |
| Bathing | 0(0-1) | 0(0-1) | 0.31 |
| Walking on level ground | 0(0-3) | 0(0-3) | 0.29 |
| Climbing stairs | 0(0-2) | 0(0-2) | 0.47 |
| Dressing | 0(0-2) | 0(0-2) | 0.26 |
| Bowel continence | 0(0-2) | 0(0-2) | 0.07 |
| Urinary continence | 0(0-2) | 0(0-2) | 0.10 |
| At discharge, Score (IQR) |  |  |  |
| Feeding | 2(0-2) | 2(0-2) | 0.01 |
| Transferring | 3(0-3) | 2(0-3) | 0.001 |
| Grooming | 1(0-1) | 0(0-1) | <0.01 |
| Toileting | 2(0-2) | 1(0-2) | <0.001 |
| Bathing | 0(0-1) | 0(0-1) | <0.01 |
| Walking on level ground | 3(0-3) | 2(0-3) | <0.001 |
| Climbing stairs | 1(0-2) | 0(0-2) | <0.01 |
| Dressing | 1(0-1) | 0(0-1) | <0.01 |
| Bowel continence | 2(0-2) | 1(0-2) | <0.01 |
| Urinary continence | 2(0-2) | 1(0-2) | 0.001 |

Abbreviations: ADL, activities of daily living; AT, antithrombin; TM, thrombomodulin; IQR, interquartile range.
